# Membrane-tethering of cytochrome *c* accelerates regulated cell death in yeast

**DOI:** 10.1038/s41419-020-02920-0

**Published:** 2020-09-05

**Authors:** Alexandra Toth, Andreas Aufschnaiter, Olga Fedotovskaya, Hannah Dawitz, Pia Ädelroth, Sabrina Büttner, Martin Ott

**Affiliations:** 1 Department of Biochemistry and Biophysics, Stockholm University, Stockholm, Svante Arrheniusväg 16, 106 91 Stockholm, Sweden; 2 Institute of Molecular Biosciences, University of Graz, Humboldtstraße 50, 8010 Graz, Austria; 3 Department of Molecular Biosciences, The Wenner-Gren Institute, Stockholm University, Svante Arrheniusväg 20C, 106 91 Stockholm, Sweden

**Keywords:** Mitochondrial proteins, Cell death, Energy metabolism

## Abstract

Intrinsic apoptosis as a modality of regulated cell death is intimately linked to permeabilization of the outer mitochondrial membrane and subsequent release of the protein cytochrome *c* into the cytosol, where it can participate in caspase activation via apoptosome formation. Interestingly, cytochrome *c* release is an ancient feature of regulated cell death even in unicellular eukaryotes that do not contain an apoptosome. Therefore, it was speculated that cytochrome *c* release might have an additional, more fundamental role for cell death signalling, because its absence from mitochondria disrupts oxidative phosphorylation. Here, we permanently anchored cytochrome *c* with a transmembrane segment to the inner mitochondrial membrane of the yeast *Saccharomyces cerevisiae*, thereby inhibiting its release from mitochondria during regulated cell death. This cytochrome *c* retains respiratory growth and correct assembly of mitochondrial respiratory chain supercomplexes. However, membrane anchoring leads to a sensitisation to acetic acid-induced cell death and increased oxidative stress, a compensatory elevation of cellular oxygen-consumption in aged cells and a decreased chronological lifespan. We therefore conclude that loss of cytochrome *c* from mitochondria during regulated cell death and the subsequent disruption of oxidative phosphorylation is not required for efficient execution of cell death in yeast, and that mobility of cytochrome *c* within the mitochondrial intermembrane space confers a fitness advantage that overcomes a potential role in regulated cell death signalling in the absence of an apoptosome.

## Introduction

Cytochrome *c* is an evolutionary highly conserved protein localized in the mitochondrial intermembrane space (IMS), which transfers electrons from cytochrome *bc*_1_ reductase (complex III) to cytochrome *c* oxidase (COX, complex IV), a reaction regarded as the rate-limiting step of mitochondrial respiration^1^. Cytochrome *c* is a water-soluble protein that can diffuse in three dimensions in the IMS, but also associates with the inner mitochondrial membrane (IMM)^2,3^. In the baker’s yeast *Saccharomyces cerevisiae*, cytochrome *c* is encoded by *CYC1* and its paralog *CYC7*, with the first accounting for 95% of total cytochrome *c* content during aerobic growth, while the latter is expressed during hypoxia^4^. Beyond its crucial role in the mitochondrial respiratory chain, cytochrome *c* is a key player during intrinsic apoptosis, a form of regulated cell death associated with mitochondrial outer membrane permeabilization^5^. In higher eukaryotes, cytochrome *c* released from the IMS into the cytosol binds to apoptotic peptidase activating factor 1 (APAF1) and pro-caspase 9 to form the apoptosome, a supermolecular complex that initiates a caspase cascade, culminating in apoptotic cell death^5,6^.

Regulated cell death is not limited to multicellular organisms but also occurs in unicellular eukaryotes (including several yeast species) and even in some prokaryotes^5^. In yeast and higher eukaryotes, programmed necrotic and apoptotic cell death subroutines have been described as regulated cell death modalities^5,7^. While sharing key features and basic components of the molecular machinery executing regulated cell death in metazoa, yeast cells also display distinct differences. The yeast genome codes for several apoptosis-related proteins, including the metacaspase Yca1^8^, the HtrA-like protease Nma111^9^ and the mitochondrial pro-apoptotic proteins Apoptosis-inducing factor Aif1^10^ and endonuclease G^11^. Yeast apoptosis can be triggered by multiple stimuli, ranging from acetic acid^12–14^, H_2_O_2_^10,15^, ethanol^16^, hypochlorous acid^17^, UV radiation^18^ and pheromones^19^ to heterologous expression of human pro-apoptotic proteins^20^. In addition, several physiological scenarios such as mating, antagonistic interaction between yeast species^21,22^, colony formation^23,24^ as well as replicative and chronological ageing^25,26^ have been shown to trigger apoptotic death of unfit or damaged cells within a yeast population^27,28^. Although the release of cytochrome *c* can be detected in several of these scenarios^12,29^, yeast cells do not contain an apoptosome, raising the intriguing question of why cytochrome *c* release occurs in this organism. Thus, the existence of cytochrome *c* release in yeast suggests that an alternative, potentially evolutionary ancient pathway for initiation of regulated cell death might exist. Indeed, when comparing the phylogenetically conserved role of cytochrome *c* in respiration and cell death between various species, it is particularly interesting that eukaryotic cells solely express soluble forms of cytochrome *c*, allowing for high mobility within the IMS. However, some bacterial species^30,31^ harbour membrane-bound cytochrome *c* variants, which mediate electron transport during respiratory growth^31^.

The exclusive presence of soluble forms of cytochrome *c* in the mitochondrial IMS of eukaryotic cells suggests that this has evolved to allow this protein to additionally participate in apoptotic cell death. To test this hypothesis, we used baker’s yeast as a model, employing an evolutionary highly conserved, robust cell death pathway that is accompanied by the release of cytochrome *c* into the cytosol, but lacking an apoptosome. We engineered a yeast strain to contain exclusively membrane-anchored cytochrome *c* and analysed its impact on mitochondrial function, ageing and cell death. Membrane anchoring of cytochrome *c* retained proper respiratory growth and correct assembly of mitochondrial respiratory chain supercomplexes, but resulted in increased cellular respiration and elevated production of reactive oxygen species (ROS). Importantly, regulated cell death, including age-dependent as well as acetic acid-induced cell death, was accelerated in this strain, demonstrating that loss of cytochrome *c* from mitochondria does not contribute to the execution of cell death in evolutionary old regulated cell death regimes.

## Material and methods

### Construction of membrane-anchored cytochrome *c*

The *CYC1* gene from *Saccharomyces cerevisiae*, with its own promoter and terminator sequences, was synthesized by GeneArt® Gene Synthesis (Thermo Fischer Scientific) and was provided in a pRS305 vector. The mitochondrial targeting segment and the transmembrane sequence from the yeast *CYB2* (lactate dehydrogenase) gene were inserted upstream of the *CYC1* coding sequence. Thereby, the transmembrane segment of Cyb2 served as membrane-anchoring part for cytochrome *c*. In addition, the linker sequence from the membrane-anchored cytochrome *c-y* of *Rhodobacter sphaeroides*^31^, was inserted upstream of the *CYC1* gene, which confers flexibility to the membrane-anchored cytochrome *c* in yeast.

This construct encoding the membrane-anchored form of cytochrome *c* (Cyc1^MA^, coding sequence of the whole construct is given in Table 1) was PCR-amplified (primer sequences: 5′- CTGTATGTATATAAAACTCTTGTTTTCTTC -3′ and 5′- AAAAATAAATAGGG ACCTAGACTTCAGGTTGTCTAACTCC -3′), and transformed into a yeast strain (obtained from Euroscarf), lacking both forms of cytochrome *c* (BY4741 (MAT**a** *his3*Δ1 *leu2*Δ0 *met15*Δ0 *ura3*Δ0 *cyc1*∆ *cyc7*∆)), where it was chromosomally integrated via homologous recombination. In this way, Cyc1^MA^ resides in the open reading frame of the autonomous *CYC1* gene, under the control of the endogenous *CYC1* promoter.

**Table 1:** Coding sequence of the membrane-anchored cytochrome *c* variant (Cyc1^MA^).

Segment of membrane anchored Cyc1^MA^	Sequence
Promoter sequence (yeast *CYC1* promoter)	5′- GTTCATTTGGCGAGCGTTGGTTGGTGGATCAAGCCCACGCGTAGGCAATCCTCGAGCAGATCCGCCAGGCGTGTATATATAGCGTGGATGGCCAGGCAACTTTAGTGCTGACACATACAGGCATATATATATGTGTGCGACGACACATGATCATATGGCATGCATGTGCTCTGTATGTATATAAAACTCTTGTTTTCTTCTTTTCTCTAAATATTCTTTCCTTATACATTAGGACCTTTGCAGCATAAATTACTATACTTCTATAGACACACAAACACAAATACACACACTAAATTAATA -3′
Mitochondrial targeting sequence and transmembrane segment (from yeast *CYB2*)	5′- ATGTTGAAATATAAACCATTGTTGAAAATTTCTAAAAATTGTGAAGCTGCTATTTTGAGAGCTTCTAAAACTAGATTGAATACTATTAGAGCTTATGGTTCTACTGTTCCAAAATCTAAATCTTTTGAACAAGATTCTAGAAAAAGAACTCAATCTTGGACTGCTTTGAGAGTTGGTGCTATTTTGGCTGCTACTTCTTCTGTTGCTTATTTGAATTGGCATAATGGT -3′
Linker sequence (from *R. sphaeroides cyt c*_*y*_)	5′- TTGTATACTACTGGTGGTGGTCATGGTGAAGATGCTGTTCAAGCTTATGTTATTGAAACTGGTGGTGGTGGTGCTGCTGAAGAAGAACCAGATGCTGAAGCTGTTCCATTTGCTGAA -3′
Yeast cytochrome *c* 1 (*CYC1*) coding sequence	5′- ATGACTGAATTCAAGGCCGGTTCTGCTAAGAAAGGTGCTACACTTTTCAAGACTAGATGTCTACAATGCCACACCGTGGAAAAGGGTGGCCCACATAAGGTTGGTCCAAACTTGCATGGTATCTTTGGCAGACACTCTGGTCAAGCTGAAGGGTATTCGTACACAGATGCCAATATCAAGAAAAACGTGTTGTGGGACGAAAATAACATGTCAGAGTACTTGACTAACCCAAAGAAATATATTCCTGGTACCAAGATGGCCTTTGGTGGGTTGAAGAAGGAAAAAGACAGAAACGACTTAATTACCTACTTGAAAAAAGCCTGTGAGTAA -3′
Terminator sequence (yeast *CYC1* terminator)	5′- ACAGGCCCCTTTTCCTTTGTCGATATCATGTAATTAGTTATGTCACGCTTACATTCACGCCCTCCTCCCACATCCGCTCTAACCGAAAAGGAAGGAGTTAGACAACCTGAAGTCTAGGTCCCTATTTATTTTTTTTAATAGTTATGTTAGTATTAAGAACGTTATTTATATTTCAAATTTTTCTTTTTTTTCTGTACAAAC -3′

### Media and culturing conditions

Cells were grown at 28 °C and 145 rpm in synthetic complete (SC) medium, containing 0.17% yeast nitrogen base (Difco, BD Biosciences), 0.5% (NH_4_)_2_SO_4_, 30 mg/l of all amino acids (except 80 mg/l histidine and 200 mg/l leucine), 30 mg/l adenine and 320 mg/l uracil with 2% d-glucose. Full media (YEPD) agar plates contained 1% yeast extract (Bacto, BD Biosciences), 2% peptone (Bacto, BD Biosciences), 4% D-glucose and 2% agar. All media were prepared with double-distilled water and were autoclaved for 25 min at 121 °C, 210 kPa. Amino acids were prepared as 10x stocks, separately sterilized and added to the media after autoclaving.

Overnight cultures were grown for 16–20 h in SC medium using glass eprouvettes and were applied for inoculation of 10 ml SC medium in baffled 100 ml Erlenmeyer flasks to an OD_600_ of 0.3. After 6 h of incubation, cells were used for experiments. Treatments with antimycin A were performed in Erlenmeyer flasks. Therefore, antimycin A was dissolved in ethanol and added to cells after 24 h with a final concentration of 50 µM. Equivalent amounts of ethanol were added to control cells. For acetic acid treatment, cells were transferred into 96-well deep-well plates (500 µl of culture per well) and acetic acid was added to a final concentration of 120 or 160 mM. Strains were incubated for 1 h at 28 °C, 1000 rpm and subsequently applied for further analysis.

### Analysis of cellular growth

Growth was analyzed with a Bioscreen C^TM^ automated microbiology growth curve analysis system (Growth Curves USA). Cells were inoculated to an OD_600_ of 0.1 in SC media with indicated carbon sources in the suppliers “honeycomb microplate” in a final volume of 250 µl media per well and OD_600_ was measured automatically every 30 min at 28 °C and shaking on maximum level. Respective media without cells was used as blank. The doubling time was calculated from growth curves during logarithmic growth phase.

### Analysis of cell death

Loss of membrane integrity as a marker of necrotic cell death was determined via propidium iodide (PI) staining as described previously^32^. In brief, ~2 × 10^6^ cells were collected by centrifugation in 96-well plates and resuspended in 250 µl phosphate buffered saline (PBS, 25 mM potassium phosphate; 0.9% NaCl; adjusted to pH 7.2) containing 100 µg/l PI. After incubation for 10 min at room temperature (RT) in the dark, cells were washed once in PBS and 30,000 cells per sample were analysed via flow cytometry (BD LSR Fortessa; BD FACSDivia software).

For discrimination between necrotic and early/late apoptotic cell death phenotypes, AnnexinV/PI co-staining was performed according to refs. ^11,33^. Therefore, ~2 × 10^7^ cells were harvested, washed once in digestion buffer (35 mM K_3_PO_4_, 0.5 mM MgCl_2_, 1.2 M sorbitol; adjusted to pH 6.8) and resuspended in 330 µl of the same buffer containing 2.5 µl Lyticase (1000 U/ml) and 15 µl Glucoronidase/Arylsulfatase (4.5 U/ml). Spheroplastation was conducted at 28 °C and 145 rpm and digestion of the cell wall was monitored microscopically (~0.5 h). Spheroplasts were carefully washed once in 500 µl digestion buffer and subsequently resuspended in 30 µl staining buffer (10 mM HEPES, 140 mM NaCl, 5 mM CaCl_2_, 0.6 M sorbitol; adjusted to pH 7.4) containing 100 µg/l PI and 2 µl Annexin-V-FLUOS reagent (Roche). After 20 min incubation at RT in the dark, 100 µl staining buffer was added per sample and transferred into 96-well plates for subsequent analysis via flow cytometry.

Clonogenic survival was evaluated as described recently^33^. In aggregate, the number of cells in culture was quantified via a CASY cell counting device (Schärfe Systems) and 500 cells were plated on YEPD agar plates. After 2 days incubation at 28 °C colony-forming units were counted.

### Measurement of oxidative stress and mitochondrial transmembrane potential

To monitor oxidative stress, the reactive oxygen species (ROS)-driven conversion of non-fluorescent dihydroethidium (DHE) to fluorescent ethidium (Eth) was quantified with flow cytometry and visualized with confocal laser scanning microscopy, adapted from ref. ^33^. To that end, ~2 × 10^6^ cells were harvested in 96-well plates, resuspended in 250 µl PBS containing 2.5 mg/l DHE and incubated for 10 min at RT in the dark. Afterwards, cells were washed and analysed as described for PI staining above.

To investigate mitochondrial transmembrane potential (Δ*ψ*_m_), the protocol described in ref. ^33^ was slightly adapted. ~2 × 10^6^ cells were resuspended in 250 µl PBS containing 5% glucose and 200 nM Mitotracker CMXRos. Cells were incubated, washed and analysed as described for DHE staining above.

### Microscopy

Specimen were prepared on agar slides to immobilize yeast cells and investigated with a Leica SP5 confocal laser scanning microscope, equipped with a Leica HCX PL Apo 63× NA 1.4 oil immersion objective. Z-stacks were acquired using 64 × 64 × 12.6 (x/y/z) nm sampling and analysed as well as processed with the open-source software Fiji^34^. To that end, three-dimensional Gaussian filtering (xσ = yσ = zσ = 1), followed by background subtraction (rolling ball radius = 50 pixels) was applied and pictures were illustrated using the maximum-intensity projection method. Volume rendering to visualize mitochondrial morphology (“Projection” in Fig. 2b, d and f) was applied with the build-in Fiji Macro “Volume Viewer” by Kai Uwe Barthel (Mode: Volume; Interpolation: Trillinear; Sampling: 1.0). For micrographs presented in Fig. 3g, samples were prepared on agar slides and analysed with a Leica DM6B epifluorescence microscope, using a HC PL Apo 100× NA 1.4 oil immersion objective. The dynamic range of presented figures was adapted by using the “Brightness/contrast” tool of Fiji. All pictures within an experiment were captured and processed with the same settings.

### Measurement of cellular oxygen consumption

Oxygen consumption of yeast was quantified with a Fire-Sting optical oxygen sensor system (Pyro Science) as described previously^33^. In brief, 2 ml of culture were transferred into glass tubes, hermetically sealed and directly used for analysis. Oxygen concentration was measured for 1 min and the slope of the regression line was calculated and normalized to the number of PI negative (and thus viable) cells in the glass tube (evaluated by CASY cell counting and quantification of PI negative cells with flow cytometry as described above). Oxygen consumption per living cells is expressed as fold value normalized to wild type cells.

### Isolation of mitochondria

Isolation of mitochondria from yeast cells was performed as described in ref. ^35^. In brief, cells grown to mid-logarithmic phase were harvested by centrifugation and resuspended in 2 ml/g cell wet weight MP1 buffer (0.1 M Tris-H_2_SO_4_, 10 mM dithiothreitol; adjusted to pH 9.4). After incubation for 10 min at 30 °C, samples were washed in 1.2 M sorbitol and resuspended in MP2 buffer (20 mM potassium phosphate, 0.6 M sorbitol; adjusted to pH 7.4), containing 3 mg/g of cell wet weight zymolyase 20 T. Spheroplasts were created by incubation for 1 h at 30 °C and harvested by centrifugation. Samples were carefully resuspended in 13.4 ml/g of cell wet weight in homogenization buffer (10 mM Tris, 0.6 M sorbitol, 1 mM EDTA, 1 mM PMSF; adjusted pH 7.4) and homogenized by 10 strokes with a Teflon plunger (Sartorius Stedim Biotech S.A.). Homogenates were centrifuged at 3000 g for 5 min at 4 °C and the resulting supernatants were subsequently centrifuged at 17,000 g for 12 min at 4 °C. Pelleted mitochondria were resuspended in isotonic buffer (20 mM HEPES, 0.6 M sorbitol; adjusted to pH 7.4) to a concentration of 10 mg/ml.

### Immunoblot analysis

To obtain whole-cell extracts, cells were harvested by centrifugation at 18,400 g for 2 min and resuspended in 50 µL of Laemmli buffer (63 mM Tris, 2% SDS, 10% glycerol, 0.1% β-mercaptoethanol and 0.0005% bromophenol blue; adjusted to pH 6.8). Samples were boiled for 3 min at 95 °C and 10 µl were applied for SDS-PAGE and immunoblotting following standard protocols.

For submitochondrial fractionation experiments, 100 µg of mitochondrial protein were treated with 0.2 M NaCl and five freeze-and-thaw cycles in liquid nitrogen were performed. After centrifugation at 100,000×*g* for 30 min at 4 °C, supernatants and pellet fractions were treated with trichloroacetic acid (12% final concentration) to precipitate proteins. Samples were incubated for 20 min at −20 °C and afterwards centrifuged at 28,000×*g* for 30 min at 4 °C. Pellet fractions were washed with acetone, followed by additional centrifugation at 28,000 rcf for 15 min at 4 °C. Of note, samples for total protein were left untreated. Finally, samples were boiled at 95 °C for 3 min, resuspended in Laemmli buffer and 10 µl of the samples were applied for SDS-PAGE and immunoblotting following standard protocols. Blots were probed with antibodies against cytochrome *c* (holo form), Tom70, Mdh1 and Aco1 as loading control, which were kindly provided by Nora Vögtle, University of Freiburg. Peroxidase-conjugated secondary anti-rabbit antibodies (BioRad, 1705046 and Sigma, A0545) were used for chemiluminescence detection.

### Blue native electrophoresis

Isolated mitochondria were centrifuged at 16,000 g for 10 min at 4 °C and the pellet was subsequently resuspended in lysis buffer (50 mM Bis-Tris, 25 mM KCl, 2 mM Aminohexanoic acid, 12% glycerol, 1 mM PMSF, 2% digitonin and Complete Protease Inhibitor cocktail (Roche). 100 µg of mitochondrial protein was loaded on a 3–12% precast native gel (Invitrogen), which was subsequently stained with Coomassie.

### UV-VIS spectroscopy

Optical spectra (350–700 nm) were recorded using Cary4000 UV-Vis spectrophotometer (Agilent Technologies). The concentration of *a*-type hemes was determined from the sodium dithionite-reduced minus potassium ferricyanide-oxidized difference spectra using the absorption coefficient *ε* (630–605 nm) = 23.2 mM^−1^ ^36^. Concentrations of *b*- and *c*-type hemes were measured simultaneously from difference spectra as described in ref. ^37^, using the following formula:

[heme *b*] (mM) = (Δ(A^562^ − A^577^) × 3.539 × 10^–2^ − Δ(A^553^ − A^540^)) × 1.713 × 10^–3^

[heme *c*] (mM) = (Δ(A^553^ − A^540^) × 5.365 × 10^–2^ − Δ(A^562^ − A^577^)) × 9.564 × 10^–3^

### Measurement of oxygen reduction rate in isolated mitochondria

Isolated mitochondria were resuspended in HEPES buffer (20 mM HEPES, 250 mM sucrose, 50 mM KCl, 0.1 mM EDTA; adjusted to pH 7.4). Cytochrome *c* oxidase activity (the oxygen reduction rate) was monitored using a Clark-type oxygen electrode (Hansatech) with 1 ml chamber volume. 10 µM antimycin A (cytochrome *bc*_1_ inhibitor) and 0.5 µM FCCP (Carbonyl cyanide-4-(trifluoromethoxy)phenylhydrazone, uncoupling agent) were added to the reaction chamber. Sodium ascorbate (5 mM) was used as electron donor, 0.5 mM TMPD (N,N,N′,N′-Tetramethyl-p-Phenylenediamine) as electron mediator. Addition of mitochondria (at a final concentration in the range of 2–5 nM cytochrome *c* oxidase) started the reaction. Coupled cytochrome *bc*_1_ / cytochrome *c* oxidase activity was measured upon addition of 0.12 mM decylhydroquinone (DBH_2_) as electron donor. Baseline oxygen-consumption (auto-oxidation of DBH_2_) was recorded before addition of the mitochondria. Oxygen consumption was blocked by addition of KCN, verifying that the observed oxygen reduction was due to the activity of cytochrome *c* oxidase. Mitochondria devoid of the mitochondrial outer membrane (OMM) were prepared as described above. Where needed, 5 µM yeast cytochrome *c* (Sigma, C2436) was added to the mixture before addition of DBH_2_. Decylubiquinone (Sigma, D7911) was reduced to DBH_2_ by addition of a small crystal of potassium borohydride to 50 µl of 60 mM decylubiquinone in DMSO. 5 µl aliquots of 0.1 M HCI were added with gentle mixing until the yellow solution became colorless. DBH_2_ was transferred to a fresh tube, avoiding borohydride crystals. Final DBH_2_ concentration was determined from the A^277 nm^ using *ε*_277_ = 16 mM^−1^. Coupled activity values were normalized to the concentration of cytochrome *c* oxidase (*a*-type heme).

### Statistics and data representation

Results are presented as line graphs or bar charts, indicating mean ± standard error of the mean (s.e.m.), or dot plots with mean (square) ± s.e.m. and median (centre line), as well as single data points. Exact sample sizes are given in the figure legends and represent biological replicates, except for Fig. 4e, where technical replicates were used. The sample size was thereby chosen according to empirical values that are standard in the field. Visualized data were taken from representative experiments that were replicated at least two times. Randomization was not performed in our study and investigators were not blinded. Outliers were identified using the 2.2-fold interquartile range labelling rule (outlier data points are highlighted in turquoise) and data was transformed upon the presence of outliers (detailed description of the method used in Supplementary Table 1; no data was excluded from the analysis). Normality of data was evaluated with a Shapiro–Wilk’s test and homogeneity of variances was examined with a Levene’s test (both analysed with Origin Pro 2018). A detailed description of the procedure upon violation of respective assumptions is given Supplementary Table 1. In brief, means of two groups were compared with a two-sample *t*-Test (with Welch correction upon the presence of significantly different variances). The means of three or more groups were compared upon the presence of one independent variable (genotype) with a One-way Analysis of Variance (ANOVA) followed by a Bonferroni post hoc test (calculated with Origin Pro 2018) or a Welch’s ANOVA with a Games-Howell post hoc test in case of significantly heterogenous variances (analysed with IBM SPSS Statistics, Version 25). To compare the means of groups upon the presence of two independent variables (genotype and treatment), a two-way ANOVA followed by a Bonferroni post hoc test was conducted with Origin Pro 2018. Analysis of cell death over time was statistically evaluated with a two-way ANOVA mixed design (strain as between-subject and time as within-subject factor) with a Bonferroni post hoc test using Origin Pro 2018. Significances for analyses with one independent variable are indicated with asterisks (***P < 0.001, **P < 0.01, *P < 0.05, n.s. *P* > 0.05), and for two independent variables main effects are displayed with diamonds (###*P* < 0.001, ##*P* < 0.01, #*P* < 0.05, n.s. *P* > 0.05), simple main effects are depicted as asterisks (***P < 0.001, **P < 0.01, *P < 0.05, n.s. *P* > 0.05). Calculated *p*-values are presented in Supplementary Table 1. All figures were created with Origin Pro 2018 and further processed with Adobe Illustrator CS6.

## Results

### Generation and characterization of a yeast model with membrane-tethered cytochrome *c*

**Fig. 1:**
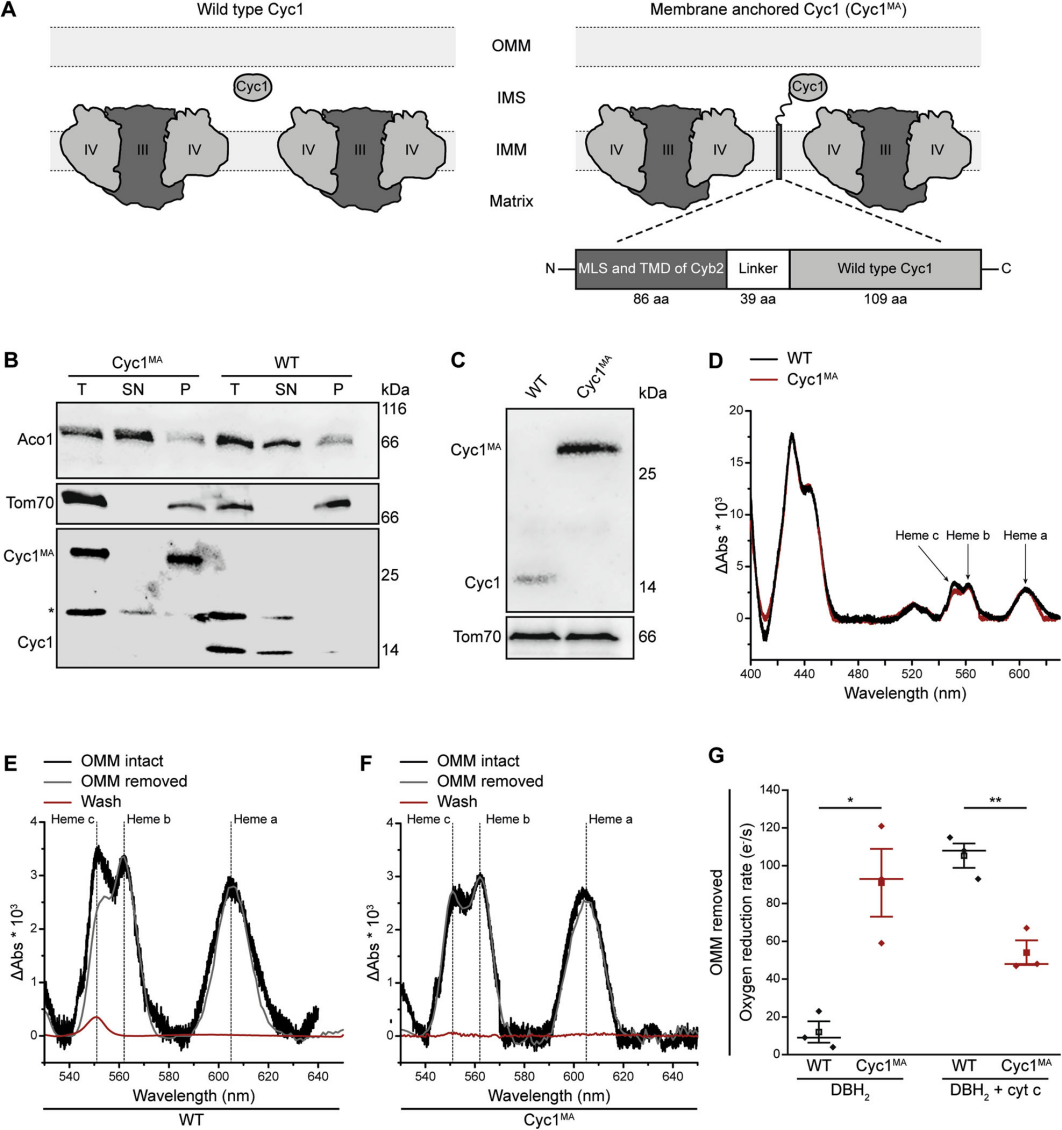
Generation of a yeast model with membrane-anchored cytochrome *c*. **a** Scheme of respiratory supercomplexes in yeast, with wild type cytochrome *c* (left panel) and genetically engineered membrane-anchored cytochrome *c* (Cyc1^MA^; right panel). For construction of Cyc1^MA^, the mitochondrial localization sequence (MLS) and the transmembrane domain (TMD) of cytochrome *b2* (Cyb2) from *Saccharomyces cerevisiae* was chromosomally integrated at the N-terminus of the *CYC1* gene together with the linker sequence of *Rhodobacter sphaeroides* cytochrome *c*_γ_. **b** Immunoblot analysis of submitochondrial fractionation with lysates from isolated mitochondria of wild type (WT) and Cyc1^MA^ cells. Total lysates (T) were separated via ultracentrifugation and the supernatant (SN) and pellet (P) fractions were applied for immunoblotting. Blots were probed with antibodies against aconitase (Aco1), Cyc1 and Tom70. The asterisk indicates a cross reaction of the Cyc1 antibody. **c** Immunoblot analysis of isolated mitochondria from WT and Cyc1^MA^ strains. Blots were probed with antibodies against Cyc1 and the outer mitochondrial membrane protein Tom70. **d**–**f** Reduced-minus-oxidized difference spectra of WT and Cyc1^MA^ strains. Spectra were normalized to the maxima of the α band at 605 nm. **d** Direct comparison of spectra from WT and Cyc1^MA^ strains. **e** Spectra from WT mitochondria before (black) and after (grey) removal of the outer mitochondrial membrane (OMM), as well as from respective wash fraction (red). **f** Spectra from Cyc1^MA^ mitochondria before (black) and after (grey) removal of the outer mitochondrial membrane (OMM), as well as from respective wash fraction (red). **g** Measurement of decylubiquinol (DBH_2_)-driven coupled *bc*_1_ complex and cytochrome *c* oxidase activity in isolated mitochondria devoid of the outer mitochondrial membrane (OMM) of WT as well as *CYC7* deletion strains harbouring a membrane-anchored form of Cyc1 (Cyc1^MA^). Where indicated, yeast cytochrome *c* (cyt *c*) was added. The oxygen reduction rate was calculated as electrons per second (e^−^/s) per cytochrome *c* oxidase. Mean (square) ± s.e.m., median (centre line) and single data points (*n* = 3) are depicted. *P < 0.05; **P < 0.01.

**Fig. 2:**
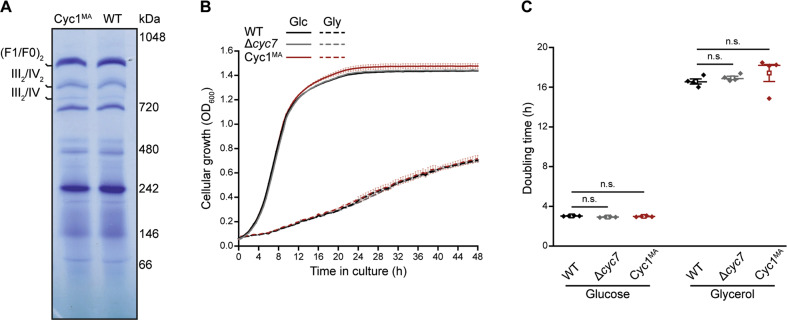
Characterization of yeast cells expressing membrane-anchored cytochrome c. **a** Blue native electrophoresis of isolated mitochondria from WT and Cyc1^MA^ strains. Gel was stained with Coomassie and respiratory supercomplexes are highlighted. **b**, **c** Cellular growth of strains described in **a**, analysed via automatic measurement of optical density (OD_600_) every 30 min with a Bioscreen C^TM^ growth curve system either on glucose (Glc) or glycerol (Gly) as carbon source, respectively. Growth curves **b** as well as doubling time in exponential growth phase **c** are presented. Line graph in **b** shows mean ± s.e.m. For box plot in **c**, mean (square) ± s.e.m., median (centre line) and single data points (*n* = 4) are depicted. n.s. not significant.

To test the significance of cytochrome *c* loss from mitochondria for rudimentary cell death regimes, we genetically engineered baker´s yeast to exclusively express a membrane-anchored form of the wild type cytochrome *c* protein (Cyc1), which we termed Cyc1^MA^. In brief, we deleted the two cytochrome *c* genes *CYC1* and *CYC7* and chromosomally integrated *CYC1*^MA^ into the original locus of *CYC1*. Thereby, *CYC1*^MA^ consists of the mitochondrial localization sequence and transmembrane domain of cytochrome *b2* (*CYB2*) from *S. cerevisiae* at the N-terminus, connected to the linker region of membrane-anchored cytochrome *c-*γ from *R. sphaeroideus* followed by the coding sequence of wild type *CYC1* (Fig. 1A; please see material and method section, as well as Table 1 for details). Submitochondrial fractionation experiments revealed the presence of Cyc1^MA^ in the membrane fraction, confirming its anchoring to the IMM, whereas wild type Cyc1 was mainly found in the soluble fraction (Fig. 1b). Immunoblotting using isolated mitochondria demonstrated increased protein levels of the engineered Cyc1^MA^ variant compared to the native Cyc1 (Fig. 1c). However, reduced-minus-oxidized difference spectra from these mitochondria showed a slight reduction of heme *c* content in cells harbouring Cyc1^MA^ compared to the wild type (Fig. 1d–f), indicating a mild decrease in hemylated cytochrome *c* upon membrane-tethering. Upon removal of the outer mitochondrial membrane (OMM), cytochrome *c* was released from wild type, as evidenced by a decrease in heme *c* content (Fig. 1e) from the treated mitochondria and the appearance of a cytochrome *c* spectrum in the wash fraction. As expected, Cyc1^MA^ was not released from the organelles (Fig. 1f), further confirming anchoring of Cyc1^MA^ in the IMM. In addition, we measured coupled *bc*_1_ / cytochrome *c* oxidase (COX) activity in isolated mitochondria devoid of the OMM. The oxygen consumption of mitochondria from wild type cells was significantly lower than that of mitochondria from the Cyc1^MA^ strain, again demonstrating the efficient retention of membrane-anchored cytochrome *c* at the IMM (Fig. 1g). Addition of yeast cytochrome *c* could recover oxygen reduction activity in wild type mitochondria, but decreased oxygen consumption in Cyc1^MA^ mitochondria (Fig. 1g), most probably due to the flow of electrons from the *bc*_1_ complex to the excess of added oxidised soluble cytochrome *c*. Next, we performed blue native PAGE of isolated mitochondria, showing correct assembly of respiratory supercomplexes in the Cyc1^MA^ strain (Fig. 2a). These results are in line with regular growth of all investigated strains on fermentable and non-fermentable media (Fig. 2b, c). In summary, we have created a yeast strain expressing a membrane-anchored variant of Cyc1, which is functional with respect to assembly of respiratory supercomplexes and growth under fermentative and non-fermentative conditions. This demonstrates that the ability of cytochrome *c* to diffuse in three dimensions in the IMS is not essential for respiration, in line with the observation that membrane-anchored cytochrome *c* variants are active in bacterial respiratory chains^31^.

### Loss of cytochrome *c* mobility causes cell death during exponential growth

**Fig. 3:**
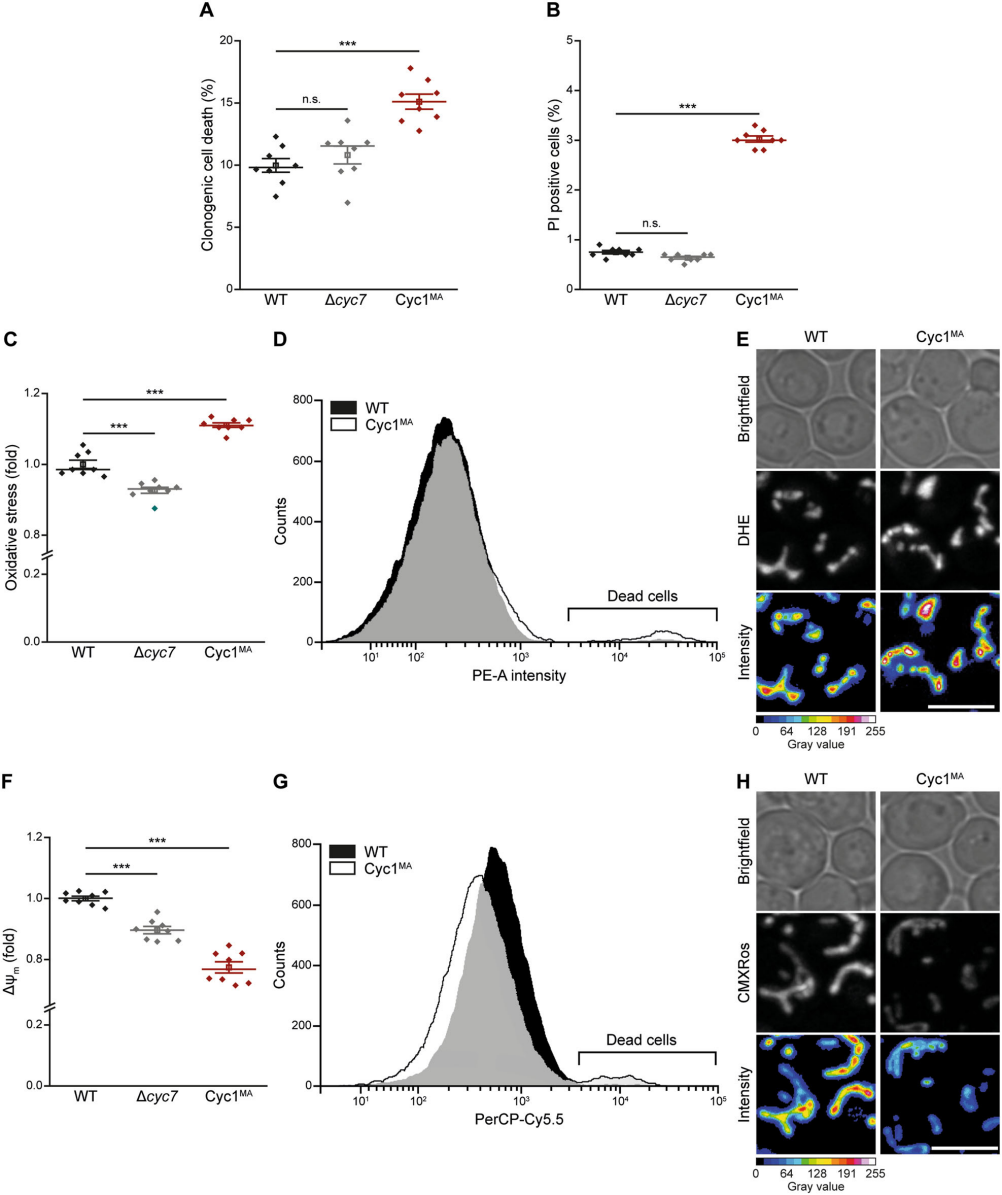
Loss of cytochrome c mobility enhances cell death of exponentially growing yeast cells. **a** Clonogenic death of wild type (WT), *CYC7* deletion (Δ*cyc7*), as well as *CYC7* deletion strains harbouring a membrane-anchored form of Cyc1 (Cyc1^MA^), determined by counting colony-forming units after plating 500 cells of indicated strains on YEPD agar plates. **b** Flow cytometric quantification of loss of membrane integrity as indicated by propidium iodide (PI) staining of cells described in **a**. **c**–**e** Analysis of oxidative stress, determined by the reactive oxygen species-driven conversion of non-fluorescent dihydroethidium (DHE) to fluorescent ethidium (Eth) of cells described in **a**. **c** Flow cytometric quantification of mean fluorescence intensities is shown as fold values of WT cells. **d** Dead cells, accumulating Eth due to a loss of membrane integrity, were excluded from the analysis. **e** Z-projections of representative confocal micrographs of DHE-stained cells are depicted. **f**–**h** Quantification and visualization of mitochondrial transmembrane potential (Δ*ψ*_m_) of cells described above. **f** Flow cytometrically quantified mean fluorescence intensities of Mitotracker CMXRos-stained cells are presented as fold values of WT cells. **g** Dead cells, accumulating the fluorescent dye due to a loss of membrane integrity were excluded from the analysis. **h** Z-projections of representative confocal micrographs of Mitotracker CMXRos-stained cells are depicted. Mean (square) ± s.e.m., median (centre line) and single data points (*n* = 8) are depicted. Data point indicated in turquoise was identified as an outlier by using the 2.2-fold interquartile range labelling rule. n.s. not significant, ***P < 0.001; scale bars represent 5 µm.

Next, we evaluated how anchoring of Cyc1 to the IMM and thus lack of its release from mitochondria affects the ability of yeast cells to undergo cell death. Determination of clonogenic survival demonstrated that Cyc1^MA^ expressing cells displayed slightly decreased viability during exponential growth compared to wild type or *CYC7* deleted cells (Fig. 3a). Additional flow cytometric quantification of PI staining, indicating loss of plasma membrane integrity and thus necrotic cell death, showed that membrane-anchoring of cytochrome *c* caused a small increase in cell death (Fig. 3b). However, clonogenic death was generally higher compared to the loss of membrane integrity, suggesting a significant proportion of non-necrotic cell death in all analysed strains. Thus, we evaluated oxidative stress, a common pre-requisite of regulated cell death^38^, by monitoring the ROS-driven conversion of non-fluorescent DHE to fluorescent ethidium (Eth). This revealed that the Cyc1^MA^ mutant accumulated increased levels of ROS (Fig. 3c, d), which apparently originate from mitochondria as indicated by confocal microscopy (Fig. 3e). In turn, mitochondrial transmembrane potential (Δ*ψ*_m_) was reduced in the Cyc1^MA^ strain compared to control cells (Fig. 3f–h), an event associated with late stages of regulated cell death in yeast^20^. Together, these results suggest that the reduction of cytochrome *c* mobility leads to slightly increased cell death in exponentially growing cells, revealing phenotypes of mitochondria-dependent regulated cell death.

### Mobility of cytochrome *c* is important to maintain yeast lifespan and resistance against regulated cell death stimuli

**Fig. 4:**
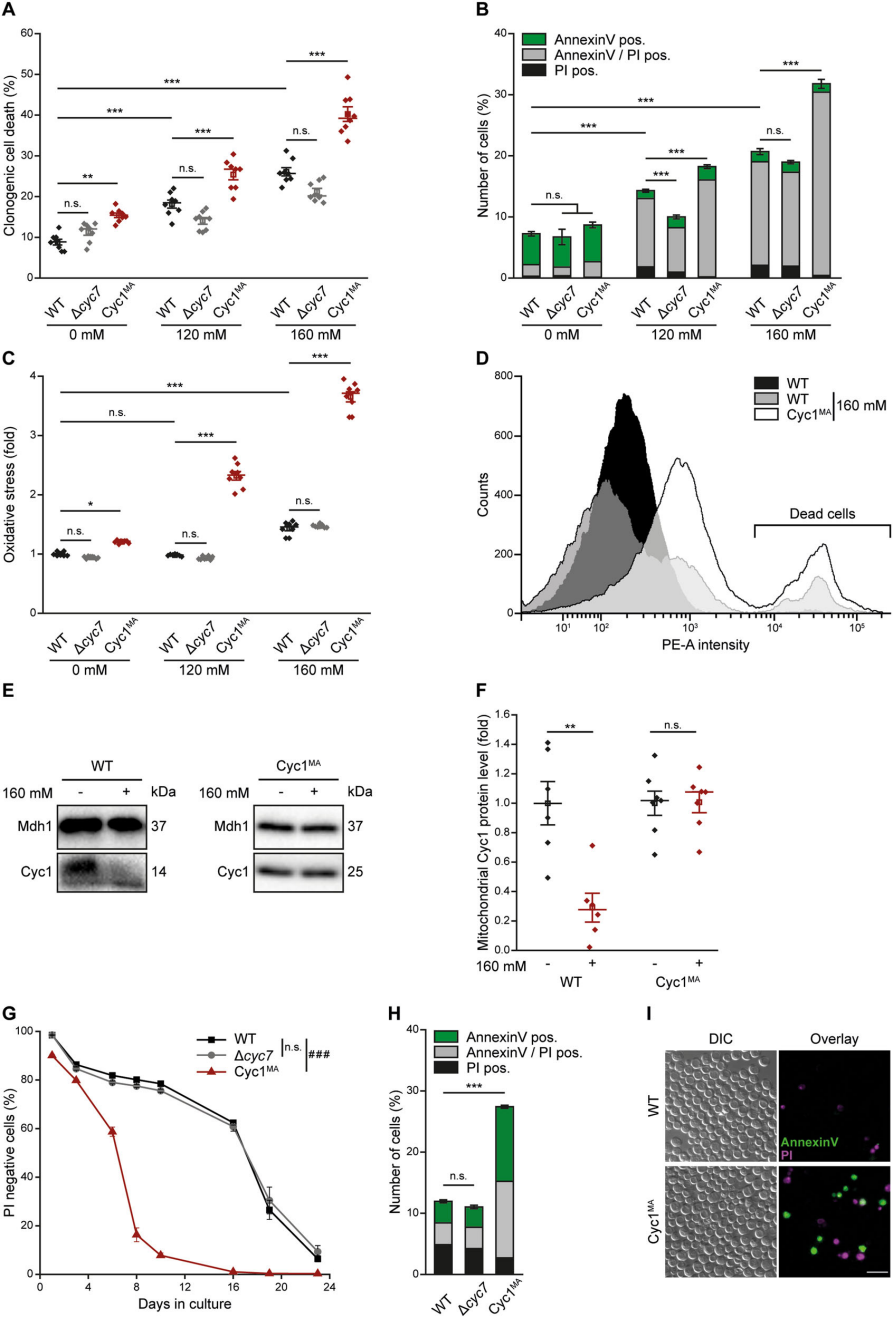
Reduced mobility of cytochrome *c* sensitizes to regulated cell death stimuli and reduces chronological lifespan of yeast. **a** Clonogenic death of wild type (WT) and *CYC7* deletion strains (Δ*cyc7*), as well as *CYC7* deletion strain harbouring a membrane-anchored form of Cyc1 (Cyc1^MA^) during exponential growth. Cells were treated with indicated concentrations of acetic acid for 1 h and colony-forming units were quantified after plating 500 cells of indicated strains on YEPD agar plates. **b** Flow cytometric quantification of AnnexinV/propidium iodide (PI) co-staining of cells described in **a**. **c**, **d** Analysis of oxidative stress, indicated by the reactive oxygen species-driven conversion of non-fluorescent dihydroethidium (DHE) to fluorescent ethidium (Eth) of cells described in **a**. **c** Flow cytometric quantification of mean fluorescence intensities is shown as fold values of WT cells. **d** Dead cells, accumulating Eth due to a loss of membrane integrity, were excluded from the analysis. **e**, **f** Immunoblot analysis of isolated mitochondria from WT and Cyc1^MA^ strains. Cells were treated with 160 mM acetic acid for 1 h directly before isolation of mitochondria (+) or left untreated (−). Blots were probed with antibodies against Cyc1 and Mdh1 (mitochondrial malate dehydrogenase) as loading control. Relative levels were calculated after normalization to Mdh1 levels. Representative immunoblots (**e**) as well as densitometric quantification of mitochondrial Cyc1 levels (**f**) are shown. **g** Chronological lifespan of indicated strains, evaluated by the flow cytometric quantification of PI negative cells. **h, i** Quantification of cell death via AnnexinV/PI co-staining of cells as described in **b** after 48 h. **h** Flow cytometric quantification and **i** representative epifluorescence micrographs are presented. Bar charts in **b**, **h** and line graph in **g** show mean ± s.e.m. (*n* = 8). For box plots, mean (square) ± s.e.m., median (centre line) and single data points (**a**, **c**: *n* = 8; **f**: *n* = 6 for wild type and *n* = 7 for Cyc1^MA^) are depicted. Simple main effects are visualized as n.s. not significant, *P < 0.05, **P < 0.01 and ***P < 0.001; main effects are presented as n.s. not significant and ^###^*P* < 0.001 in **g**; scale bar indicates 10 µm.

Due to these surprising effects of Cyc1^MA^ on cell death and oxidative stress during normal growth, we next investigated the response to stimuli triggering regulated cell death. Treatment with acetic acid is a regime frequently applied to induce this cell death subroutine in yeast^11–13^ and results in a dose-dependent reduction of viability. Anchoring of Cyc1 to the IMM caused a clear increase in clonogenic cell death (Fig. 4a). To test for phosphatidylserine externalisation, an early apoptotic event, we performed AnnexinV/PI co-staining, which allows discrimination between early apoptotic (AnnexinV pos.), late apoptotic/secondary necrotic (AnnexinV/PI pos.) and primary necrotic (PI pos.) cell death phenotypes^7,29^. The Cyc1^MA^ mutant showed significantly increased rates of late apoptotic/secondary necrotic cell death phenotypes (Fig. 4b). High levels of ROS upon acetic acid treatment were detectable in the Cyc1^MA^ mutant (Fig. 4c, d), likely explaining higher sensitivity of this strain against regulated cell death stimuli. Dead cells randomly accumulating the dye due to a loss of membrane integrity have been excluded from the analysis (Fig. 4d). To confirm the loss of cytochrome *c* from wild type mitochondria upon acetic acid treatment, as well as the absence of this process in the Cyc1^MA^ mutant, cytochrome *c* protein levels were evaluated in isolated mitochondria of each strain, comparing cells that received acetic acid treatment 1 h prior to mitochondrial preparation and respective untreated controls. While the mitochondrial levels of wild type Cyc1 were prominently reduced in treated cells, levels of Cyc1^MA^ were not changed (Fig. 4e, f), owing to its membrane anchoring (Fig. 1b, f). Furthermore, determination of chronological lifespan revealed premature death of the Cyc1^MA^ during ageing (Fig. 4g). This was at least in part due to an induction of apoptosis, as we observed a prominent increase in phosphatidylserine externalisation in the Cyc1^MA^ mutant after 48 h (Fig. 4h, i). Together, these results demonstrate that membrane-anchoring of cytochrome *c* increases sensitivity to cell death stimuli, elevates age-dependent regulated cell death and hence reduces the chronological lifespan of yeast cells. This suggests that the loss of cytochrome *c* from mitochondria is not required for cell death signalling in cells lacking an apoptosome. In contrast, anchoring cytochrome *c* as an integral inner membrane protein decreases cellular stress resistance.

### Alterations of cytochrome *c* mobility dysregulates mitochondrial function

**Fig. 5:**
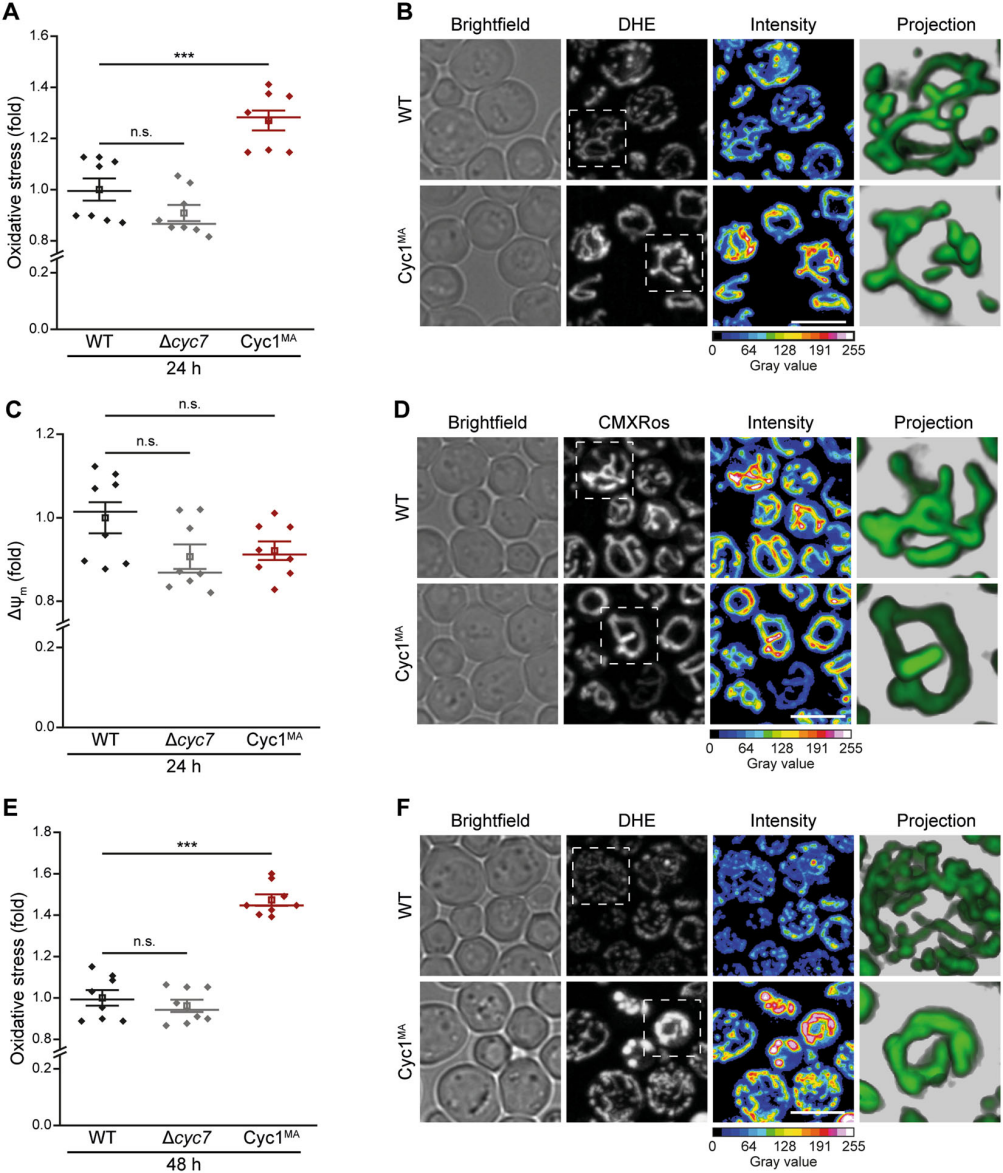
Stationary yeast cells with membrane-tethered cytochrome c show altered mitochondrial morphology and increased oxidative stress. **a**, **b** Analysis of oxidative stress, determined by the reactive oxygen species-driven conversion of non-fluorescent dihydroethidium (DHE) to fluorescent ethidium (Eth) of wild type (WT) and *CYC7* deletion strains (Δ*cyc7*), as well as *CYC7* deletion strain harbouring a membrane-anchored form of Cyc1 (Cyc1^MA^) after 24 h. **a** Flow cytometric quantification of mean fluorescence intensities is shown as fold values of WT cells. Dead cells, accumulating Eth due to a loss of membrane integrity, were excluded from the analysis. **b** Z-projections of representative confocal micrographs of DHE-stained cells are depicted. **c**, **d** Determination of mitochondrial transmembrane potential (Δψ_m_) via Mitotracker CMXRos staining of cells as described above. **c** Flow cytometrically quantified mean fluorescence intensity is presented as fold values of WT cells. Dead cells, accumulating the fluorescent dye, due to a loss of membrane integrity, were excluded from the analysis. **d** Z-projections of representative confocal micrographs of Mitotracker CMXRos-stained cells are depicted. For analysis of Δ*ψ*_m_ after 48 h, please see Supplementary Fig. 1. **e**, **f** Evaluation of oxidative stress as described in **a** after 48 h in culture. **e** Flow cytometric quantification and **f** representative confocal micrographs are shown. Mean (square) ± s.e.m., median (centre line) and single data points (*n* = 8) are depicted. n.s. not significant, ***P < 0.001; scale bars indicate 5 µm.

Prior to entering stationary phase, yeast cells undergo physiological changes during the diauxic shift, in which the metabolism changes from fermentation to respiration, resulting in increased mitochondrial abundance^39^. To test whether changes in mitochondrial functionality precede the premature death of Cyc1^MA^ cells during chronological ageing, we analysed the impact of Cyc1 anchoring on mitochondrial transmembrane potential, morphology and oxidative stress in stationary phase. Compared to exponentially growing cells (Fig. 3c–e), oxidative stress further increased after 24 h in the Cyc1^MA^ strain (Fig. 5a). Microscopic analysis revealed mitochondria as the main source of ROS and, in addition, unveiled a diminished branching of the mitochondrial network compared to wild type cells (Fig. 5b). Even though Δψ_m_ was stabilized in stationary phase after 24 h (Fig. 5c, d) and 48 h (Supplementary Fig. 1), alterations in mitochondrial morphology could be confirmed with this staining approach, indicating lower branching and partially circularization of mitochondria (Fig. 5d). While ROS production further increased after 48 h in the Cyc1^MA^ strain compared to control cells (Fig. 5e, f), the mitochondrial morphology appeared quite heterogenous in the Cyc1^MA^ strain, with circular mitochondria showing the highest ROS levels and mitochondria similar to that in wild type cells, however, still displaying accumulation of ROS (Fig. 5f). In sum, our data demonstrates increased oxidative stress in chronologically aged Cyc1^MA^ cells, most likely being causative for the observed increase of regulated cell death and resulting decreased chronological lifespan.

### Enhanced cellular respiration is a compensatory mechanism to counteract toxicity caused by reduced respiratory chain activity

**Fig. 6:**
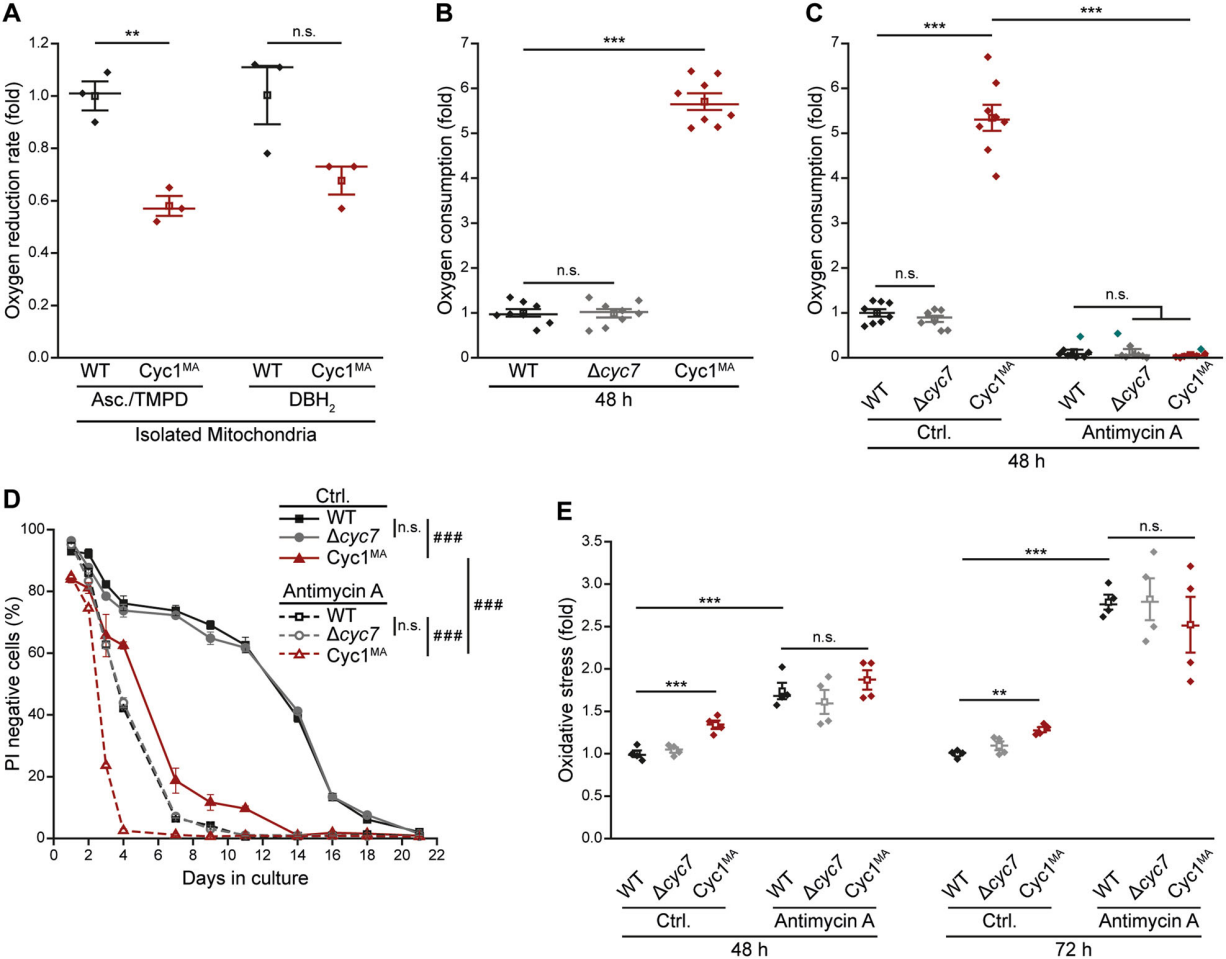
Reduced mobility of cytochrome *c* decreases respiratory chain activity and induces compensatory upregulation of respiration. **a** Measurement of Ascorbate (Asc) and N,N,N′,N′-tetremethyl-p-phenylenediamine hydrochloride (TMPD)-driven cytochrome *c* oxidase (COX) activity and decylubiquinol (DBH_2_)-driven coupled *bc*_1_ complex and COX activity in isolated mitochondria of logarithmically growing wild type (WT) cells as well as the *CYC7* deletion strain harbouring a membrane-anchored form of Cyc1 (Cyc1^MA^). Activity was calculated as electron per second per COX and was normalized to WT cells in order to present the oxygen reduction rate as fold value. **b** Cellular oxygen consumption of WT, *CYC7* deletion (Δ*cyc7)* and Cyc1^MA^ strains after 48 h. Measured oxygen consumption was normalized to living cells and subsequently presented as fold of WT cells. **c** Measurement of cellular oxygen consumption as described in **b**. Cells were treated after 24 h with 50 µM antimycin A (dissolved in ethanol) and with equivalent amounts of ethanol as a control (Ctrl.). Normalization was performed as described above. **d** Chronological lifespan of indicated strains, evaluated by the flow cytometric quantification of PI negative cells. Cells were treated with antimycin A as described in **c**. **e** Analysis of oxidative stress, determined by the reactive oxygen species-driven conversion of non-fluorescent dihydroethidium (DHE) to fluorescent ethidium (Eth) of cells treated with antimycin A and respective controls as described in **c**. Measurement was performed after 48 and 72 h. Mean (square) ± s.e.m., median (centre line) and single data points (*n* = 3 in **a** and *n* = 8 in **b**, **c**) are depicted for dot plots in (**a**–**c**, **e**). Data points indicated in turquoise were identified as outliers by using the 2.2-fold interquartile range labelling rule. Line graph in **d** shows mean ± s.e.m. (*n* = 4). Simple main effects are visualized as n.s. not significant, **P < 0.01 and ***P < 0.001; Main effects are presented as n.s. not significant and ^###^*P* < 0.001.

Finally, we investigated if alterations in the mitochondrial respiratory chain might be causative for the observed increase in oxidative stress. Since we detected normal assembly of respiratory chain supercomplexes in the Cyc1^MA^ strain (Fig. 2a), we analysed enzymatic activity of their components. COX activity was measured by supplying ascorbate (Asc) and N,N,N′,N′-tetremethyl-p-phenylenediamine hydrochloride (TMPD), as well as carbonyl cyanide-4-(trifluoromethoxy)phenylhydrazone (FCCP) as uncoupling agent to isolated mitochondria, and was significantly higher in wild type cells compared to the Cyc1^MA^ strain (Fig. 6a). Also coupled *bc*_1_ complex / COX activity (assessed with decylubiquinol; DBH_2_) was reduced in the Cyc1^MA^ strain (Fig. 6a), presumably both due to the somewhat reduced levels of hemylated cytochrome *c* in the membrane-anchored variant. Since Δ*ψ*_m_ was only reduced in exponential but not in stationary Cyc1^MA^ cells (Fig. 3f–h and 5c, d and Supplemenatry Fig. 1), we hypothesized that yeast cells compensate this reduced respiratory chain activity during ageing. Hence, we analysed cellular oxygen consumption in chronologically aged yeast cell cultures and observed a massive upregulation of respiration in the Cyc1^MA^ strain compared to control cells after 48 h (Fig. 6b). To evaluate if this respiratory upregulation was a cytoprotective mechanism to compensate decreased respiratory chain activity, we inhibited this response by administration of antimycin A to stationary cultures after 24 h^40^, and again measured cellular oxygen consumption after 48 h, confirming inhibition of respiration by this treatment (Fig. 6c).

While the antimycin A-induced inhibition of respiration reduced the lifespan of all strains tested (Fig. 6d), this effect was most prominent in Cyc1^MA^ cells. Disrupting respiration drastically increased the premature death of cells harbouring membrane-tethered cytochrome *c*, clearly indicating that the viability of these cells depends on the observed upregulation of respiratory activity (Fig. 6d). In addition, we observed increased accumulation of ROS upon antimycin A treatment. However, no difference in oxidative stress between Cyc1^MA^ cells and respective controls were detectable upon antimycin A treatment, while we again observed increased ROS levels in the untreated Cyc1^MA^ mutant compared to wild type cells (Fig. 6e). Thus, antimycin A induced oxidative stress in all strains, but selectively triggered cell death of Cyc1^MA^ cells during early days of ageing (Fig. 6d, e). Hence, antimycin A does not reduce the lifespan of Cyc1^MA^ cells via an additional burst of oxidative stress, but rather by inhibiting respiration in these cells.

In aggregate, our results demonstrate that diminished cytochrome *c* mobility results in reduced respiratory chain activity, leading to an upregulation of cellular respiration that sustains viability.

## Discussion

Beyond its biological function as an electron carrier in respiratory chains, cytochrome *c* is a key regulator of apoptotic cell death. Its release into the cytosol and the subsequent formation of an apoptosome triggers the activation of a caspase cascade^6^. Release of cytochrome *c* from mitochondria also occurs during regulated cell death in yeast^12,41–43^, an organism lacking an orthologous apoptosome. This evolutionary conserved cytochrome *c* release therefore could reflect a rudimentary form of signalling that could drive regulated cell death signalling in largely diverse eukaryotes. Possible scenarios are that either the depletion of cytochrome *c* from mitochondria, which subsequently impairs oxidative phosphorylation, or its presence in the cytosol modulates cell death. Likewise, complete lack of active cytochrome *c*, either via deletion of the genes coding for the two cytochrome *c* isoforms or via deletion of *CYC3*, encoding the cytochrome *c* heme lyase, has been shown to confer tolerance to distinct regulated cell death stimuli, including acetic acid^12^, manganese^44^ or human lactoferrin^45^ in yeast. Here, we report that anchoring cytochrome *c* as an integral membrane protein to the IMM and thus preventing its loss from mitochondria does not inhibit regulated cell death. These data therefore conclusively demonstrate that reduction of cytochrome *c* in mitochondria is not a causal event of regulated cell death signalling in cases where an apoptosome is missing. On the contrary, cells harbouring membrane-anchored cytochrome *c* were sensitised to regulated cell death stimuli with phenotypes of mitochondria-related cell death^20^ showing altered mitochondrial morphology, increased respiration, transient impairment of the Δ*ψ*_m_ and increased ROS production. These cellular changes ultimately result in early cell death with apoptotic phenotypes and a reduced chronological lifespan.

How could permanent membrane tethering of cytochrome *c* decrease cellular fitness? Despite a mild reduction in coupled *bc*_1_ complex/COX activity in the Cyc1^MA^ mutant, we observed increased cellular respiration of this strain, indicating a compensatory mechanism. This increase potentially elevates ROS levels, which we detected in the Cyc1^MA^ variant. Nevertheless, an inhibition of this response via antimycin A treatment drastically decreased the lifespan of Cyc1^MA^ strains compared to wild type cells. This was not simply due to a further accumulation of ROS, as we did not observe any additional accumulation of oxidative stress in Cyc1^MA^ cells treated with antimycin A compared to wild type cells receiving the same treatment. Instead, our data demonstrate that this upregulation of respiration in the Cyc1^MA^ strain represents a compensatory mechanism that sustains viability during ageing.

The molecular mechanism resulting in ROS production in Cyc1^MA^ strains remains to be further investigated. However, it was suggested in both mammalian^46^ and yeast cells^47^ that the release of cytochrome *c* into the cytosol might participate in a defence mechanism against oxidative stress by acting as a ROS scavenger. Hence, inhibition of this release from mitochondria might increase oxidative stress, resulting in cytochrome *c*-release-independent cell death, a form of regulated cell death described for yeast cells before^48^. Interestingly, during initial stages of apoptosis, cytochrome *c* has been shown to interact with and to selectively peroxidize cardiolipin in the IMM^49,50^. This peroxidation weakens the binding of cytochrome *c* to cardiolipin and facilitates its detachment from the IMM and subsequent release into the cytosol^2,50^. The peroxidase activity of cytochrome *c* is highly dependent on distinct surface charges facilitating interaction and structural arrangements upon binding to cardiolipin^51,52^. Thus, it is likely that anchoring cytochrome *c* to the IMM favours its peroxidase activity, leading to a progressive increase of peroxidized cardiolipin that likely sensitizes cells towards cell death induction. A high degree of cardiolipin peroxidation not only facilitates the release of mitochondrial apoptogenic factors besides cytochrome *c*^50^, but has also been shown to reduce cytochrome *c* oxidase activity^53^. In this line, we find a modest decrease in coupled *bc*_1_ complex/COX activity, which might be explained by potential alterations of cytochrome *c* peroxidase activity upon its membrane anchoring, subsequently affecting COX enzymatic activity.

The W65S mutation in yeast cytochrome *c* was demonstrated to abolish both its release into the cytosol and regulated cell death^54^, which might contradict our study. However, the authors of this study state that this mutation impairs electron transfer to COX and prevents respiratory growth. The Cyc1^MA^ strain showed a slight decrease in coupled *bc*_1_ complex/COX activity, which might be caused by alterations of cytochrome *c* peroxidase activity as described above or by the mild reduction of hemylated cytochrome *c* in these cells. Importantly, this effect was very mild compared to the W65S mutations, since no alteration of growth was observed in Cyc1^MA^ strains, neither on fermentable nor on non-fermentable carbon sources. These differences are further emphasized by the fact that the W65S mutation caused decreased oxidative stress^54^, while the membrane-anchoring of cytochrome *c* elevated ROS levels.

While mitochondrial cytochrome *c* is a soluble mobile electron carrier, it occurs as both a soluble (Cyt *c-2*) or a membrane bound (Cyt *c-y*) form of cytochrome *c*^55^ in alpha-proteobacteria, the evolutionary ancestor of mitochondria. Certain alpha-proteobacteria can carry out both aerobic and anaerobic respiration as well as photosynthesis. For example, *Rhodobacter sphaeroides* employs the soluble Cyt *c-2* in photosynthesis, while the membrane bound form operates in respiratory electron transfer^55^. The adverse effects of membrane-tethered cytochrome *c* as revealed in this study suggests that soluble cytochrome *c* variants have been favoured during evolution, so that membrane tethered cytochrome *c* variants no longer operate as mobile electron carriers in mitochondria.

In sum, we show that preventing the loss of cytochrome *c* from mitochondria by tethering it to the IMM increases sensitivity towards acetic acid and exacerbates regulated cell death during ageing, indicating that the release of cytochrome *c* is not required for the induction of regulated cell death in yeast. Hence, a scenario where cytochrome *c* loss from mitochondria and the subsequent inhibition of oxidative phosphorylation is essential for regulated cell death appears to be unlikely. It is tempting to speculate that control of regulated cell death via cytochrome *c* mobility in the mitochondrial IMS and potentially cardiolipin peroxidation as an early event in regulated cell death is a phylogenetically older mode of this cell death routine, which was subsequently complemented by apoptosome-dependent regimes in higher eukaryotes to execute apoptosis. The strategy employed here to tether cytochrome *c* to the IMM could be used to investigate the consequences of absence of cytochrome *c* release in mammalian apoptosis.

## Supplementary information

Supplementary Figure Legends

Supplementary Figure 1

Supplementary Table 1
