# Supplementary material for: Membrane-tethering of cytochrome c accelerates regulated cell death in yeast
Source: Cell Death Dis. 2020 Sep 5;11(9):722. doi: 10.1038/s41419-020-02920-0 (PMC7474732; doi:10.1038/s41419-020-02920-0)
Supplement: Supplementary file 1 — Supplementary Figure Legends [file 41419_2020_2920_MOESM1_ESM.docx]

**Supplementary Figure 1: Mitochondrial transmembrane potential is not altered in stationary yeast cells with membrane-anchored cytochrome c.**

Flow cytometric quantification of mitochondrial transmembrane potential (Δψ_m_) of wild type (WT) and *CYC7* deletion strains (Δ*cyc7*), as well as *CYC7* deletion strains harbouring a membrane-anchored form of Cyc1 (Cyc1^MA^) after 48 h. Mean fluorescence intensity of Mitotracker CMXRos-stained cells are presented as fold values of WT cells. Dead cells, accumulating the fluorescent dye due to loss of membrane integrity, were excluded from analysis.

Mean (square) ± s.e.m., median (centre line) and single data points (n=8) are depicted. n.s. not significant.
