# Supplementary figures and images for: Membrane-tethering of cytochrome c accelerates regulated cell death in yeast
Source: Cell Death Dis. 2020 Sep 5;11(9):722. doi: 10.1038/s41419-020-02920-0 (PMC7474732; doi:10.1038/s41419-020-02920-0)

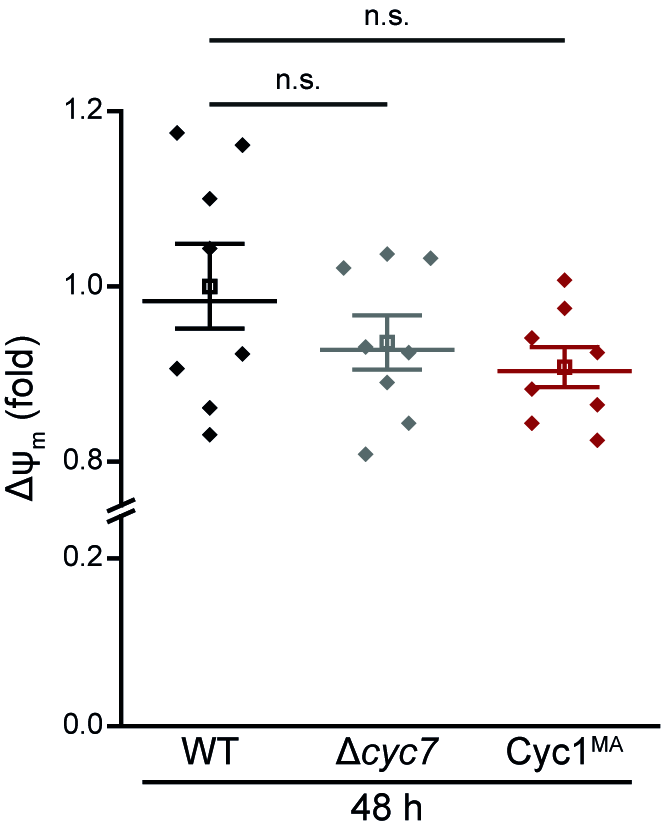

Supplement: Supplementary file 2 — Supplementary Figure 1 [file 41419_2020_2920_MOESM2_ESM.tif]
