# Supplementary material for: Membrane-tethering of cytochrome c accelerates regulated cell death in yeast
Source: Cell Death Dis. 2020 Sep 5;11(9):722. doi: 10.1038/s41419-020-02920-0 (PMC7474732; doi:10.1038/s41419-020-02920-0)
Supplement: Supplementary file 3 — Supplementary Table 1 [file 41419_2020_2920_MOESM3_ESM.docx]

**Supplementary Table 1: Detailed description of statistical analyses performed in this study.**

| **Figure** | **Statistical test** | | **Additional information** | | | **p-values** |
| --- | --- | --- | --- | --- | --- | --- |
| 1 G | Two-sample t-Test | | All assumptions met for both treatments | | | Upon DBH_2_ 0.014  Upon DBH_2_ + cyt c 0.005 |
| 2 C | One-Way ANOVA with Bonferroni post hoc test | | Data not normally distributed and significantly different variances detected; therefore, data transformed with log(max(X)+1-(X)) for statistical analysis; | | | WT Glc vs. Δ*cyc7* Glc 1.000  WT Glc vs. Cyc1^MA^ Glc 1.000  Cyc1^MA^ Glc vs. Δ*cyc7* Glc 1.000  WT Gly vs. Δ*cyc7* Gly 1.000  WT Gly vs. Cyc1^MA^ Gly 0.273  Cyc1^MA^ Gly vs. Δ*cyc7* Gly 0.949  WT Glc vs. WT Gly 0.000  Δ*cyc7* Glc vs. Δ*cyc7* Gly 0.000  Cyc1^MA^ Glc vs. Cyc1^MA^ Gly 0.000  WT Glc vs. Δ*cyc7* Gly 0.000  WT Glc vs. Cyc1^MA^ Gly 0.000  WT Gly vs. Δ*cyc7* Glc 0.000  WT Gly vs. Cyc1^MA^ Glc 0.000  Δ*cyc7* Gly vs. . Cyc1^MA^ Glc 0.000  Δ*cyc7* Glc vs. . Cyc1^MA^ Gly 0.000 |
| 3 A | One-Way ANOVA with Bonferroni post hoc test | | All assumptions met | | | WT vs. Δ*cyc7*: 1.000  WT vs. Cyc1^MA^: 0.000  Δ*cyc7* vs. Cyc1^MA^: 0.000 |
| 3 B | One-Way ANOVA with Bonferroni post hoc test | | Test conducted despite non-normally distributed data due to identical sample size (n=8) | | | WT vs. Δ*cyc7*: 0.239  WT vs. Cyc1^MA^: 0.000  Δ*cyc7* vs. Cyc1^MA^: 0.000 |
| 3 C | One-Way ANOVA of transformed data with Bonferroni post hoc test | | Outlier identified (marked in turquoise); therefore, data transformed with log((max(X)+1)-X) for statistical analysis | | | WT vs. Δ*cyc7*: 0.000  WT vs. Cyc1^MA^: 0.000  Δ*cyc7* vs. Cyc1^MA^: 0.000 |
| 3 F | Welch-ANOVA with Games-Howell post hoc test | | Significantly different variances detected; therefore, a Welch-ANOVA was used for statistical analysis | | | WT vs. Δ*cyc7*: 0.000  WT vs. Cyc1^MA^: 0.000  Δ*cyc7* vs. Cyc1^MA^: 0.000 |
| 4 B | Two-Way ANOVA with Bonferroni post hoc test | | Total amount of dead cells was analysed. Test conducted despite significantly different variances due to identical sample size (n=8). Significant interaction between strains and treatment detected. Significances of simple main effects visualized with * | | | Main effects for strain:  WT vs. Δ*cyc7*: 1.000  WT vs. Cyc1^MA^: 0.002  Δ*cyc7* vs. Cyc1^MA^: 0.001  Main effects for treatment:  0 mM vs. 120 mM: 1.000  0 mM vs. 160 mM: 0.000  120 mM vs. 160 mM: 0.000  Simple main effects for interaction:  WT 0 mM vs. WT 120 mM: 0.000  WT 0 mM vs. WT 160 mM: 0.000  WT 120 mM vs. WT 160 mM: 0.000  WT 0 mM vs. Δ*cyc7* 0 mM: 1.000  WT 0 mM vs. Δ*cyc7* 120 mM: 0.051  WT 0 mM vs. Δ*cyc7* 160 mM: 0.000  WT 120 mM vs. Δ*cyc7* 0 mM: 0.000  WT 120 mM vs. Δ*cyc7* 120 mM: 0.000  WT 120 mM vs. Δ*cyc7* 160 mM: 0.000  WT 160 mM vs. Δ*cyc7* 0 mM: 0.000  WT 160 mM vs. Δ*cyc7* 120 mM: 0.000  WT 160 mM vs. Δ*cyc7* 160 mM: 1.000  WT 0 mM vs. Cyc1^MA^ 0 mM: 1.000  WT 0 mM vs. Cyc1^MA^ 120 mM: 0.000  WT 0 mM vs. Cyc1^MA^ 160 mM: 0.000  WT 120 mM vs. Cyc1^MA^ 0 mM: 0.000  WT 120 mM vs. Cyc1^MA^ 120 mM: 0.000  WT 120 mM vs. Cyc1^MA^ 160 mM: 0.000  WT 160 mM vs. Cyc1^MA^ 0 mM: 0.000  WT 160 mM vs. Cyc1^MA^ 120 mM: 0.143  WT 160 mM vs. Cyc1^MA^ 160 mM: 0.000  Δ*cyc7* 0 mM vs. Δ*cyc7* 120 mM: 0.007  Δ*cyc7* 0 mM vs. Δ*cyc7* 160 mM: 0.000  Δ*cyc7* 120 mM vs. Δ*cyc7* 160 mM: 0.000  Δ*cyc7* 0 mM vs. Cyc1^MA^ 0 mM: 0.728  Δ*cyc7* 0 mM vs. Cyc1^MA^ 120 mM: 0.000  Δ*cyc7* 0 mM vs. Cyc1^MA^ 160 mM: 0.000  Δ*cyc7* 120 mM vs. Cyc1^MA^ 0 mM: 1.000  Δ*cyc7* 120 mM vs. Cyc1^MA^ 120 mM: 0.000  Δ*cyc7* 120 mM vs. Cyc1^MA^ 160 mM: 0.000  Δ*cyc7* 160 mM vs. Cyc1^MA^ 0 mM: 0.000  Δ*cyc7* 160 mM vs. Cyc1^MA^ 120 mM: 1.000  Δ*cyc7* 160 mM vs. Cyc1^MA^ 160 mM: 0.000  Cyc1^MA^ 0 mM vs. Cyc1^MA^ 120 mM: 0.000  Cyc1^MA^ 0 mM vs. Cyc1^MA^ 160 mM: 0.000  Cyc1^MA^ 120 mM vs. Cyc1^MA^ 160 mM: 0.000 |
| 4 A | Two-Way ANOVA with Bonferroni post hoc test | | Test conducted despite significantly different variances due to identical sample size (n=8). Significant interaction between strains and treatment detected. Significances of simple main effects visualized with * | | | Main effects for strain:  WT vs. Δ*cyc7*: 0.042  WT vs. Cyc1^MA^: 0.000  Δ*cyc7* vs. Cyc1^MA^: 0.000  Main effects for treatment:  0 mM vs. 120 mM: 0.000  0 mM vs. 160 mM: 0.000  120 mM vs. 160 mM: 0.000  Simple main effects for interaction:  WT 0 mM vs. WT 120 mM: 0.000  WT 0 mM vs. WT 160 mM: 0.000  WT 120 mM vs. WT 160 mM: 0.000  WT 0 mM vs. Δ*cyc7* 0 mM: 1.000  WT 0 mM vs. Δ*cyc7* 120 mM: 0.031  WT 0 mM vs. Δ*cyc7* 160 mM: 0.000  WT 120 mM vs. Δ*cyc7* 0 mM: 0.001  WT 120 mM vs. Δ*cyc7* 120 mM: 0.263  WT 120 mM vs. Δ*cyc7* 160 mM: 1.000  WT 160 mM vs. Δ*cyc7* 0 mM: 0.000  WT 160 mM vs. Δ*cyc7* 120 mM: 0.000  WT 160 mM vs. Δ*cyc7* 160 mM: 0.058  WT 0 mM vs. Cyc1^MA^ 0 mM: 0.001  WT 0 mM vs. Cyc1^MA^ 120 mM: 0.000  WT 0 mM vs. Cyc1^MA^ 160 mM: 0.000  WT 120 mM vs. Cyc1^MA^ 0 mM: 0.000  WT 120 mM vs. Cyc1^MA^ 120 mM: 0.000  WT 120 mM vs. Cyc1^MA^ 160 mM: 0.000  WT 160 mM vs. Cyc1^MA^ 0 mM: 0.000  WT 160 mM vs. Cyc1^MA^ 120 mM: 1.000  WT 160 mM vs. Cyc1^MA^ 160 mM: 0.000  Δ*cyc7* 0 mM vs. Δ*cyc7* 120 mM: 1.000  Δ*cyc7* 0 mM vs. Δ*cyc7* 160 mM: 0.000  Δ*cyc7* 120 mM vs. Δ*cyc7* 160 mM: 0.000  Δ*cyc7* 0 mM vs. Cyc1^MA^ 0 mM: 0.281  Δ*cyc7* 0 mM vs. Cyc1^MA^ 120 mM: 0.000  Δ*cyc7* 0 mM vs. Cyc1^MA^ 160 mM: 0.000  Δ*cyc7* 120 mM vs. Cyc1^MA^ 0 mM: 1.000  Δ*cyc7* 120 mM vs. Cyc1^MA^ 120 mM: 0.000  Δ*cyc7* 120 mM vs. Cyc1^MA^ 160 mM: 0.000  Δ*cyc7* 160 mM vs. Cyc1^MA^ 0 mM: 0.009  Δ*cyc7* 160 mM vs. Cyc1^MA^ 120 mM: 0.213  Δ*cyc7* 160 mM vs. Cyc1^MA^ 160 mM: 0.000  Cyc1^MA^ 0 mM vs. Cyc1^MA^ 120 mM: 0.000  Cyc1^MA^ 0 mM vs. Cyc1^MA^ 160 mM: 0.000  Cyc1^MA^ 120 mM vs. Cyc1^MA^ 160 mM: 0.000 |
| 4 C | | Two-Way ANOVA with Bonferroni post hoc test | | Test conducted despite significantly different variances due to identical sample size (n=8). Significant interaction between strains and treatment detected. Significances of simple main effects visualized with * | Main effects for strain:  WT vs. Δ*cyc7*: 1.000  WT vs. Cyc1^MA^: 0.000  Δ*cyc7* vs. Cyc1^MA^: 0.000  Main effects for treatment:  0 mM vs. 120 mM: 0.000  0 mM vs. 160 mM: 0.000  120 mM vs. 160 mM: 0.000  Simple main effects for interaction:  WT 0 mM vs. WT 120 mM: 1.000  WT 0 mM vs. WT 160 mM: 0.000  WT 120 mM vs. WT 160 mM: 0.000  WT 0 mM vs. Δ*cyc7* 0 mM: 1.000  WT 0 mM vs. Δ*cyc7* 120 mM: 1.000  WT 0 mM vs. Δ*cyc7* 160 mM: 0.000  WT 120 mM vs. Δ*cyc7* 0 mM: 1.000  WT 120 mM vs. Δ*cyc7* 120 mM: 1.000  WT 120 mM vs. Δ*cyc7* 160 mM: 0.000  WT 160 mM vs. Δ*cyc7* 0 mM: 0.000  WT 160 mM vs. Δ*cyc7* 120 mM: 0.000  WT 160 mM vs. Δ*cyc7* 160 mM: 1.000  WT 0 mM vs. Cyc1^MA^ 0 mM: 0.020  WT 0 mM vs. Cyc1^MA^ 120 mM: 0.000  WT 0 mM vs. Cyc1^MA^ 160 mM: 0.000  WT 120 mM vs. Cyc1^MA^ 0 mM: 0.007  WT 120 mM vs. Cyc1^MA^ 120 mM: 0.000  WT 120 mM vs. Cyc1^MA^ 160 mM: 0.000  WT 160 mM vs. Cyc1^MA^ 0 mM: 0.007  WT 160 mM vs. Cyc1^MA^ 120 mM: 0.000  WT 160 mM vs. Cyc1^MA^ 160 mM: 0.000  Δ*cyc7* 0 mM vs. Δ*cyc7* 120 mM: 1.000  Δ*cyc7* 0 mM vs. Δ*cyc7* 160 mM: 0.000  Δ*cyc7* 120 mM vs. Δ*cyc7* 160 mM: 0.000  Δ*cyc7* 0 mM vs. Cyc1^MA^ 0 mM: 0.001  Δ*cyc7* 0 mM vs. Cyc1^MA^ 120 mM: 0.000  Δ*cyc7* 0 mM vs. Cyc1^MA^ 160 mM: 0.000  Δ*cyc7* 120 mM vs. Cyc1^MA^ 0 mM: 0.000  Δ*cyc7* 120 mM vs. Cyc1^MA^ 120 mM: 0.000  Δ*cyc7* 120 mM vs. Cyc1^MA^ 160 mM: 0.000  Δ*cyc7* 160 mM vs. Cyc1^MA^ 0 mM: 0.001  Δ*cyc7* 160 mM vs. Cyc1^MA^ 120 mM: 0.000  Δ*cyc7* 160 mM vs. Cyc1^MA^ 160 mM: 0.000  Cyc1^MA^ 0 mM vs. Cyc1^MA^ 120 mM: 0.000  Cyc1^MA^ 0 mM vs. Cyc1^MA^ 160 mM: 0.000  Cyc1^MA^ 120 mM vs. Cyc1^MA^ 160 mM: 0.000 | |
| 4 F | | Two-sample t-Test | | All assumptions met | WT 0 mM (-) vs. WT 160 mM (+) 0.002  Cyc1^MA^ 0 mM (-) vs. Cyc1^MA^ 160 mM (+): 0.953 | |
| 4 G | | Two-Way ANOVA mixed design with Bonferroni post hoc test | | Strain was considered as between-subject factor, time as within-subject factor; Significant interaction between strains and time detected. Significances of main effects visualized with #. | Main effects for strain:  WT vs. Δ*cyc7*: 1.000  WT vs. Cyc1^MA^: 0.000  Δ*cyc7* vs. Cyc1^MA^: 0.000 | |
| 4 H | | One-Way ANOVA with Bonferroni post hoc test | | All assumptions met | WT vs. Δ*cyc7*: 0.063  WT vs. Cyc1^MA^: 0.000  Δ*cyc7* vs. Cyc1^MA^: 0.000 | |

| 5 A | One-Way ANOVA with Bonferroni post hoc test | Test conducted despite non-normally distributed data due to identical sample size (n=8) | WT vs. Δ*cyc7*: 0.316  WT vs. Cyc1^MA^: 0.000  Δ*cyc7* vs. Cyc1^MA^: 0.000 |
| --- | --- | --- | --- |
| 5 C | Welch-ANOVA with Games-Howell post hoc test | Significantly different variances detected; therefore, a Welch-ANOVA was used for statistical analysis | WT vs. Δ*cyc7*: 0.159  WT vs. Cyc1^MA^: 0.206  Δ*cyc7* vs. Cyc1^MA^: 0.922 |
| 5 E | One-Way ANOVA with Bonferroni post hoc test | All assumptions met | WT vs. Δ*cyc7*: 1.000  WT vs. Cyc1^MA^: 0.000  Δ*cyc7* vs. Cyc1^MA^: 0.000 |
| 6 A | Two-sample t-Test | All assumptions met for Asc./TMPD treatment;  For DBH_2_ treatment, test conducted despite non-normally distributed data due to identical sample size (n=3) | Upon Asc./TMPD: 0.003  Upon DBH_2_ 0.058 |
| 6 B | Welch-ANOVA with Games-Howell post hoc test | Significantly different variances detected; therefore, a Welch-ANOVA was used for statistical analysis | WT vs. Δ*cyc7*: 0.997  WT vs. Cyc1^MA^: 0.000  Δ*cyc7* vs. Cyc1^MA^: 0.000 |
| 6 C | Two-Way ANOVA with Bonferroni post hoc test | Outliers identified (marked in turquoise); therefore, data transformed with sqrt(X) for statistical analysis; Significant interaction between strains and treatment detected. Significances of simple main effects visualized with * | Main effects for strain:  WT vs. Δ*cyc7*: 1.000  WT vs. Cyc1^MA^: 0.000  Δ*cyc7* vs. Cyc1^MA^: 0.000  Main effects for treatment:  Ctrl. vs. Antimycin A 0.000  Simple main effects for interaction:  WT Ctrl. vs. Δ*cyc7* Ctrl.: 1.000  WT Ctrl. vs. Cyc1^MA^ Ctrl.: 0.000  Δ*cyc7* Ctrl. vs. Cyc1^MA^ Ctrl.: 0.000  WT Ctrl. vs. WT Antimycin A: 0.000  WT Ctrl. vs. Δ*cyc7* Antimycin A: 0.000  WT Ctrl. vs. Cyc1^MA^ Antimycin A: 0.000  Δ*cyc7* Ctrl. vs. WT Antimycin A: 0.000  Δ*cyc7* Ctrl. vs. Δ*cyc7* Antimycin A: 0.000  Δ*cyc7* Ctrl. vs. Cyc1^MA^ Antimycin A: 0.000  Cyc1^MA^ Ctrl. Vs. WT Antimycin A: 0.000  Cyc1^MA^ Ctrl. Vs. Δ*cyc7* Antimycin A: 0.000  Cyc1^MA^ Ctrl. Vs. Cyc1^MA^ Antimycin A: 0.000  WT Antimycin A vs.  Δ*cyc7* Antimycin A: 1.000  WT Antimycin A vs.  Cyc1^MA^ Antimycin A: 1.000  Δ*cyc7*Antimycin A vs.  Cyc1^MA^ Antimycin A: 1.000 |

| 6 D | | Two-Way ANOVA mixed design with Bonferroni post hoc test | Condition was considered as between-subject factor, time as within-subject factor; Significant interaction between condition and time detected.  Test conducted despite significantly different variances, due to identical sample size (n=4) Significances of main effects visualized with #. | | Main effects for condition:  WT Ctrl. vs. Δ*cyc7* Ctrl*.*: 1.000  WT Ctrl. vs. Cyc1^MA^ Ctrl.: 0.000  Δ*cyc7* Ctrl. vs. Cyc1^MA^ Ctrl: 0.000  WT Antimycin A vs. Δ*cyc7* Antimycin A: 1.000  WT Antimycin A vs. Cyc1^MA^ Antimycin A: 0.000  Δ*cyc7* Antimycin A vs. Cyc1^MA^ Antimycin A: 0.000  WT Ctrl. vs. WT Antimycin A: 0.000  WT Ctrl. vs. Δ*cyc7* Antimycin A: 0.000  WT Ctrl. vs. Cyc1^MA^ Antimycin A: 0.000  WT Antimycin A vs. Δ*cyc7* Ctrl.: 0.000  WT Antimycin A vs. Cyc1^MA^ Ctrl.: 0.017  Δ*cyc7* Ctrl. vs. Δ*cyc7* Antimycin A: 0.000  Δ*cyc7* Ctrl. vs. Cyc1^MA^ Antimycin A: 0.000  Δ*cyc7* Antimycin A vs. Cyc1^MA^ Ctrl.: 0.014  Cyc1^MA^ Ctrl vs Cyc1^MA^ Antimycin A: 0.000 | |
| --- | --- | --- | --- | --- | --- | --- |
| 6 E  48 h | Two-Way ANOVA with Bonferroni post hoc test | | | Significantly different variances detected; therefore, data transformed with 1/(X) for statistical analysis; Significant interaction between strains and treatment detected. Significances of simple main effects visualized with * | | Main effects for strain:  WT vs. Δ*cyc7*: 1.000  WT vs. Cyc1^MA^: 0.003  Δ*cyc7* vs. Cyc1^MA^: 0.002  Main effects for treatment:  Ctrl. vs. Antimycin A 0.000  Simple main effects for interaction:  WT Ctrl. vs. Δ*cyc7* Ctrl.: 1.000  WT Ctrl. vs. Cyc1^MA^ Ctrl.: 0.002  Δ*cyc7* Ctrl. vs. Cyc1^MA^ Ctrl.: 0.011  WT Ctrl. vs. WT Antimycin A: 0.000  WT Ctrl. vs. Δ*cyc7* Antimycin A: 0.000  WT Ctrl. vs. Cyc1^MA^ Antimycin A: 0.000  Δ*cyc7* Ctrl. vs. WT Antimycin A: 0.000  Δ*cyc7* Ctrl. vs. Δ*cyc7* Antimycin A: 0.000  Δ*cyc7* Ctrl. vs. Cyc1^MA^ Antimycin A: 0.000  Cyc1^MA^ Ctrl. vs. WT Antimycin A: 0.068  Cyc1^MA^ Ctrl. vs. Δ*cyc7* Antimycin A: 0.642  Cyc1^MA^ Ctrl. vs. Cyc1^MA^ Antimycin A: 0.013  WT Antimycin A vs. Δ*cyc7* Antimycin A: 1.000  WT Antimycin A vs. Cyc1^MA^ Antimycin A: 1.000  Δ*cyc7*Antimycin A vs. Cyc1^MA^ Antimycin A: 1.000 |
| 6 E  72 h | Two-Way ANOVA with Bonferroni post hoc test | | | Significantly different variances detected; therefore, data transformed with 1/(X) for statistical analysis; Significant interaction between strains and treatment detected. Significances of simple main effects visualized with * | | Main effects for strain:  WT vs. Δ*cyc7*: 0.796  WT vs. Cyc1^MA^: 0.082  Δ*cyc7* vs. Cyc1^MA^: 0.684  Main effects for treatment:  Ctrl. vs. Antimycin A 0.000  Simple main effects for interaction:  WT Ctrl. vs. Δ*cyc7* Ctrl.: 1.000  WT Ctrl. vs. Cyc1^MA^ Ctrl.: 0.003  Δ*cyc7* Ctrl. vs. Cyc1^MA^ Ctrl.: 0.137  WT Ctrl. vs. WT Antimycin A: 0.000  WT Ctrl. vs. Δ*cyc7* Antimycin A: 0.000  WT Ctrl. vs. Cyc1^MA^ Antimycin A: 0.000  Δ*cyc7* Ctrl. vs. WT Antimycin A: 0.000  Δ*cyc7* Ctrl. vs. Δ*cyc7* Antimycin A: 0.000  Δ*cyc7* Ctrl. vs. Cyc1^MA^ Antimycin A: 0.000  Cyc1^MA^ Ctrl. vs. WT Antimycin A: 0.000  Cyc1^MA^ Ctrl. vs. Δ*cyc7* Antimycin A: 0.000  Cyc1^MA^ Ctrl. vs. Cyc1^MA^ Antimycin A: 0.000  WT Antimycin A vs. Δ*cyc7* Antimycin A: 1.000  WT Antimycin A vs. Cyc1^MA^ Antimycin A: 1.000  Δ*cyc7*Antimycin A vs. Cyc1^MA^ Antimycin A: 1.000 |
| Suppl. Fig. 1 | Welch-ANOVA with Games-Howell post hoc test | | | Significantly different variances detected; therefore, a Welch-ANOVA was used for statistical analysis | | WT vs. Δ*cyc7*:  WT vs. Cyc1^MA^:  Δ*cyc7* vs. Cyc1^MA^: |
